# Prospective motion correction for $R_{2}^{*}$ and susceptibility mapping using spherical navigators

**DOI:** 10.1002/mrm.30385

**Published:** 2024-12-03

**Authors:** Miriam Hewlett, Omer Oran, Junmin Liu, Maria Drangova

**Affiliations:** ^1^ Robarts Research Institute The University of Western Ontario London Ontario Canada; ^2^ Department of Medical Biophysics The University of Western Ontario London Ontario Canada; ^3^ Siemens Healthcare Limited Oakville Ontario Canada

**Keywords:** $R_{2}^{*}$ mapping, brain, navigators, prospective motion correction, susceptibility mapping

## Abstract

**Purpose:**

To perform prospective motion correction (PMC) for improved $R_{2}^{*}$ and susceptibility mapping using a purely navigator‐based approach.

**Methods:**

Spherical navigators (SNAVs) were combined with an additional FID readout for simultaneous measurement of motion and zeroth‐order field shifts. The resulting FIDSNAVs were interleaved for PMC of a multi‐echo gradient echo sequence with retrospective $B_{0}$ correction. Experiments were performed on a 3T scanner with a 32‐channel head coil. Performance was assessed in five volunteers with motion prompts derived from real unintentional motion trajectories.

**Results:**

At short TEs, PMC alone was sufficient to achieve good image quality; at longer TEs, retrospective $B_{0}$ correction was often just as important for artifact reduction as motion correction. Both PMC and retrospective $B_{0}$ correction reduced error in $R_{2}^{*}$ and susceptibility maps for all participants. Residual artifacts were observed in the most severe motion case.

**Conclusion:**

Combining SNAVs with an additional FID readout enables simultaneous motion and field correction with no additional hardware requirements, improving the fidelity of quantitative mapping in the presence of motion.

## 1 INTRODUCTION

Multi‐echo gradient echo (GRE) has a wide range of applications, with various postprocessing methods enabling an assortment of quantitative mapping techniques, ranging from Dixon MRI^1^ to $R_{2}^{*}$ relaxometry^2^ and QSM.^3^ Notably, brain iron quantification using $R_{2}^{*}$ and/or QSM has gained traction as a potential biomarker in Parkinson's disease^4^, ^5^, ^6^ and other movement disorders.^7^ In this context, it is important to consider that motion is a frequent complication during MR imaging, with as many as one in five exams requiring a repeat scan due to motion artifacts.^8^ Furthermore, motion has also been demonstrated to bias quantitative MRI biomarkers ranging from functional connectivity metrics^9^, ^10^, ^11^ to gray matter volume estimates.^12^ In such cases, this bias may be counteracted using motion correction strategies.^13^

Although there exist many retrospective motion correction approaches, prospective motion correction (PMC), consisting of real‐time updates to the imaging FOV, is a favorable approach to artifact prevention because it precludes additional spin‐history effects, maintains adequate sampling, and requires little to no modification of the image reconstruction, maintaining clinically feasible reconstruction times.^14^, ^15^ To inform updates to the imaging acquisition, real‐time motion estimates can be obtained using external monitoring systems in a sequence‐independent manner and with high temporal resolution. For example, optical tracking has been demonstrated for PMC of multiparametric mapping with applications including $R_{2}^{*}$ ^16^ and QSM.^17^ However, PMC alone does not inherently account for motion‐induced field inhomogeneities, which introduce additional phase inconsistencies. Such effects result in residual artifacts which become especially apparent at the long TEs required for $R_{2}^{*}$ mapping and QSM, but can be compensated either prospectively or retrospectively using field estimates.^18^, ^19^, ^20^ For example, optical tracking systems for PMC can be supplemented with FID navigators for retrospective $B_{0}$ correction, with applications including $R_{2}^{*}$ mapping.^21^

In fact, volumetric navigators (VNAVs) acquired within the MRI scan itself can be used to derive both motion and field change estimates using complex low‐resolution images.^22^, ^23^, ^24^ Although VNAVs offer lower temporal resolution than external monitoring systems, they are advantageous for more widespread application in that they require no additional hardware or patient handling considerations. Given their relatively long acquisition and processing times (hundreds of milliseconds), VNAVs were first applied for prospective motion and field correction in long TR applications such as single voxel spectroscopy,^25^ DTI,^26^ and CEST MRI.^27^ More recently, highly accelerated VNAVs have been used for combined motion and field correction of multi‐echo GRE for $R_{2}^{*}$ mapping and QSM^28^; however, this work was concerned with retrospective correction in which case navigator processing time is not of much concern.

To enable shorter acquisition and processing times for prospective correction, motion and field changes can be estimated directly from k‐space data using k‐space navigators, which leverage the properties of the Fourier transform under a rigid motion assumption. A variety of k‐space navigator trajectories have been employed for PMC with field estimation and correction.^29^, ^30^, ^31^ In this study, we employ spherical navigators (SNAVs) for PMC because they are robust against larger motions (up to 6°) using a relatively short reference scan.^32^ While SNAVs have never been used directly for field estimation, preliminary investigation has demonstrated that incorporating an additional FID readout (combination denoted as FIDSNAVs) enables correction of zeroth‐order field shifts.^33^, ^34^ Expanding on earlier work,^35^ we demonstrate FIDSNAVs for PMC with additional retrospective $B_{0}$ correction applied to multi‐echo GRE for $R_{2}^{*}$ mapping and QSM. We also demonstrate the benefit to fat fraction (FF) maps, which are produced as part of the same postprocessing pipeline.^36^

## 2 METHODS

### 2.1 Sequence implementation

**FIGURE 1:**
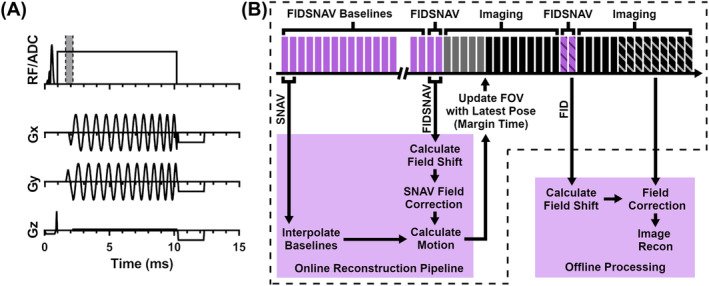
(A) FIDSNAV readout block for a single hemisphere; data acquired within the shaded region (between the FID and SNAV readouts) are discarded. (B) Outline of the imaging sequence (not to scale), along with processing pipelines for PMC and retrospective field correction. Events within the dotted line occur on the scanner during the imaging session. Although not indicated in the diagram for simplicity, field shifts were calculated relative to a reference FID. FID, free induction decay; PMC, prospective motion correction; SNAV, spherical navigator.

To avoid a purely linear kz trajectory, thus preventing the entanglement of linear phase ramps due to $B_{0}$ inhomogeneity with those resulting from translation, SNAVs were acquired as two hemispheres.^32^ The navigator sequence diagram for a single hemisphere is given in Figure 1A; x, y, and z axes correspond to the right–left, posteroanterior, and superoinferior directions assuming head‐first supine positioning. To enable zeroth‐order field monitoring (decoupled from motion estimation), an additional FID was incorporated immediately following the refocusing lobe of the slice‐selective gradient prior to SNAV acquisition (64 points with 10 μs dwell time).

These FIDSNAVs were interleaved within a 3D multi‐echo GRE sequence, as illustrated in Figure 1B, matching TR to maintain steady state.^32^, ^37^ For real‐time motion estimation, an additional reference scan consisting of pre‐rotated FIDSNAV baselines was also acquired to enable a fast lookup table approach to rotation estimation.^38^ In the implementation used for this work, only the imaging trajectory was prospectively updated; navigators interleaved throughout imaging were acquired with a constant trajectory. As such, the FIDSNAV baselines were acquired with rotated trajectories about kx, ky between [−6°,6°] in 1° increments, which is expected to cover the full range of subject motion in the majority of cases.^39^ To enable prospective updates to imaging acquisition, sequence events were prepared with real‐time sequence processing using a margin time of 10 ms. In other words, the slab positioning for each TR was calculated 10 ms prior to its planned acquisition time using the latest motion estimates, calculation of which is described in the following section.

### 2.2 Navigator processing

For efficient calculation and communication of motion estimates for PMC, real‐time processing was performed on the scanner using a custom reconstruction pipeline (overview in Figure 1B). As described in the previous section, motion estimation was accelerated using data obtained in an additional reference scan. These supplementary FIDSNAV acquisitions, performed with rotated trajectories about the kx and ky axes, were further interpolated in the latitude–longitude plane to produce a lookup table of expected SNAV signals for 3D rotations in the range of [−6°,6°] in 0.25° increments. Notably, this form of simulation by interpolation, rather than simulation by rotated trajectory, is more robust for kz rotations because they are less affected by signal changes due to $T_{2}^{*}$ decay and other factors.^32^

Prior to motion estimation, navigators interleaved throughout image acquisition were corrected using FID‐based zeroth‐order field estimates. To eliminate phase accumulation from unrelated sources, including eddy currents,^40^ each FID was first normalized to a reference FID and field shifts for data acquired prior to this point assumed to be 0 Hz. Moreover, the first 15 points of each FID were discarded due to nonlinear phase behavior. Having performed these adjustments, linear fitting of FID phase was performed on a channel‐wise basis to estimate zeroth‐order field shifts. These field estimates were then used to perform channel‐wise correction of SNAV phase prior to motion estimation in a manner similar to that described in section 2.5.

Given that rigid rotations and translations appear in k‐space as rotations and phase ramps, respectively, we first determine rotation by minimizing the magnitude residual sum‐of‐squares between the SNAV data and the lookup table entries.^32^ Phase differences between the SNAV and its best‐matched baseline scan were then unwrapped^41^ and used to estimate translation as described by Welch et al.^42^ A more detailed description of the motion estimation process can be found in a previous publication.^32^ Resulting motion parameters were then returned to the sequence for PMC as described in section 2.1.

### 2.3 Scan protocol

All experiments were performed on a 3T scanner (Magnetom PrismaFit, Siemens Healthcare GmbH, Erlangen, Germany, operating software version VE11C) using the product 32‐channel head coil. Imaging was performed using a 10‐echo protocol for multiparametric mapping based on a previous work^36^: FOV 25.6 × 25.6 × 19.2 cm, resolution 1.14 × 1.14 × 2 mm, TR 51 ms, TE 3.28/4.72/6.22/7.72/9.49/16.75/23.90/31.10/38.16/45.40 ms, flip angle 15°, receiver bandwidth 1015 Hz/pixel, acceleration factor *R* = 2 with integrated reference scan. Flow compensation was performed for the first echo only. Motion measurement accuracy with this imaging protocol was found to be similar to that determined in a previous work^34^ (section S1).

Without navigation, scan time for the imaging protocol was 10:07. Navigation was performed with FIDSNAVs interleaved at a frequency of 1.96 Hz (acquired every eight k‐space lines), in which case the eighth navigator acquisition was found to be a reliable reference for FID correction. Choosing an early reference point was required to enable real‐time field correction of SNAV data; however, some time was given to allow for stabilization of eddy current effects following the start of imaging acquisition. The increase in imaging scan time due to navigator acquisition was 2:32, with the reference scan requiring an additional 17 s. Including the additional dummy pulses, acquired to achieve steady state prior to baseline acquisition (duration 7 s), the total scan time increase was just under 30%.

### 2.4 Volunteer experiments

For preliminary demonstration, we assess the performance of this technique in a healthy volunteer study (six volunteers, age range 22–58 years, two male) approved by the Western University Health Sciences Research Ethics Board.

To assess the potential for image degradation due to navigator acquisition or correction, one volunteer was scanned with no intentional motion under the following paradigm. An initial reference scan (acquired with the unnavigated sequence) was followed by three repetitions of the following three scans: an unnavigated scan providing a measure of interscan variability, a navigated scan with no correction, and a navigated scan with PMC, all performed with randomized scan ordering.

The remaining five volunteers were scanned with intentional motion to assess the performance of motion and field correction. In the majority of cases, normal padding was employed within the head coil. To test robustness to larger motions, one subject (case 5) was scanned with reduced padding. All scans were acquired with navigators interleaved to enable comparison of motion trajectories. Following an initial reference scan with no motion and no correction, subsequent acquisitions were performed with random ordering (unknown to the participant): a scan with motion but no correction, a scan with motion and PMC processed with and without additional $B_{0}$ correction, and a repetition of the initial reference scan. To generate somewhat repeatable and realistic trajectories, participant motion was guided using a visual indicator projected into the scanner^43^ with crosshair movement based on real unintentional motion cases from an open source database.^44^ To simplify comparison of the resulting traces, movement throughout each scan was also summarized using an RMS motion score based a measure of total displacement as described by Tisdall et al.^45^ To reduce the relative weighting of slow drifts, common even for scans with no intentional motion, values of rotation and translation were taken relative to those occurring at the midpoint of the scan (coinciding with central k‐space acquisition).

### 2.5 Retrospective field correction

For fair comparison of images with and without field correction, all volunteer scans were reconstructed offline with the same custom pipeline (implemented in MatLab, Natick, MA, software version R2020b). For each navigator, zeroth‐order field shifts for each channel, $∆f_{0,\text{coil}}$, were estimated with respect to the reference FID as described in section 2.2 Navigator processing (Figure 1B). The raw imaging signal was then corrected on a channel‐wise basis using $∆f_{0,\text{coil}}$ from the nearest navigator acquisition according to,

$$S_{\text{coil}}^{B_{0,\text{corr}}}=S_{\text{coil}}\exp\left(-i2\pi ∆f_{0,\text{coil}}t\right),$$

where $S_{\text{coil}}$ denotes the raw signal for a single k‐space line, coil, with or without field correction, $B_{0,\text{corr}}$, and t the time following RF excitation. Performing the field measurement and correction for each coil individually allows some degree of spatial variation in field fluctuations to be compensated based on differences in coil sensitivity. Preliminary investigation found that correction using these channel‐wise estimates, similar to that performed in a prior work,^21^ provided improved image quality compared to that performed using a single field estimate obtained after coil combination (results not shown). Undersampled acquisitions were then reconstructed using GRAPPA.^46^, ^47^ To reduce the relative weighting of channels experiencing rapid signal loss (typically those with large motion‐induced field shifts), magnitude images were obtained using root‐sum‐of‐squares coil combination. Phase data for quantitative mapping was combined using coil sensitivities from ESPIRiT calibration^48^ as implemented in the Berkeley Advanced Reconstruction Toolbox.^49^

### 2.6 Quantitative parameter mapping

Complex images from the first five echoes were used to compute FF as described in Ref. 52 based on multiparameter fitting of the six‐peak fat model, including $B_{0}$ inhomogeneity and $R_{2}^{*}$ relaxation.^51^ Having computed FF in this manner, magnitude images from all echoes were fitted to the magnitude term of this same signal model to generate an $R_{2}^{*}$ map.^36^ For QSM, a frequency map was first generated from nonlinear least squares fitting of the complex, multi‐echo data as described in Ref. 52. Background field was removed using the Laplacian boundary value method^53^ and susceptibility maps obtained using morphology‐enabled dipole inversion.^52^

### 2.7 Image quality assessment

In all cases, image and quantitative mapping quality was assessed following registration to the initial reference scan and skull stripping (to reduce error from nonrigid motion of the ears or other). Multi‐echo, magnitude images were assessed using normalized RMSE (NRMSE) and structural similarity, whereas quantitative mapping quality was quantified using mean absolute error across the brain volume.

## 3 RESULTS

**FIGURE 2:**
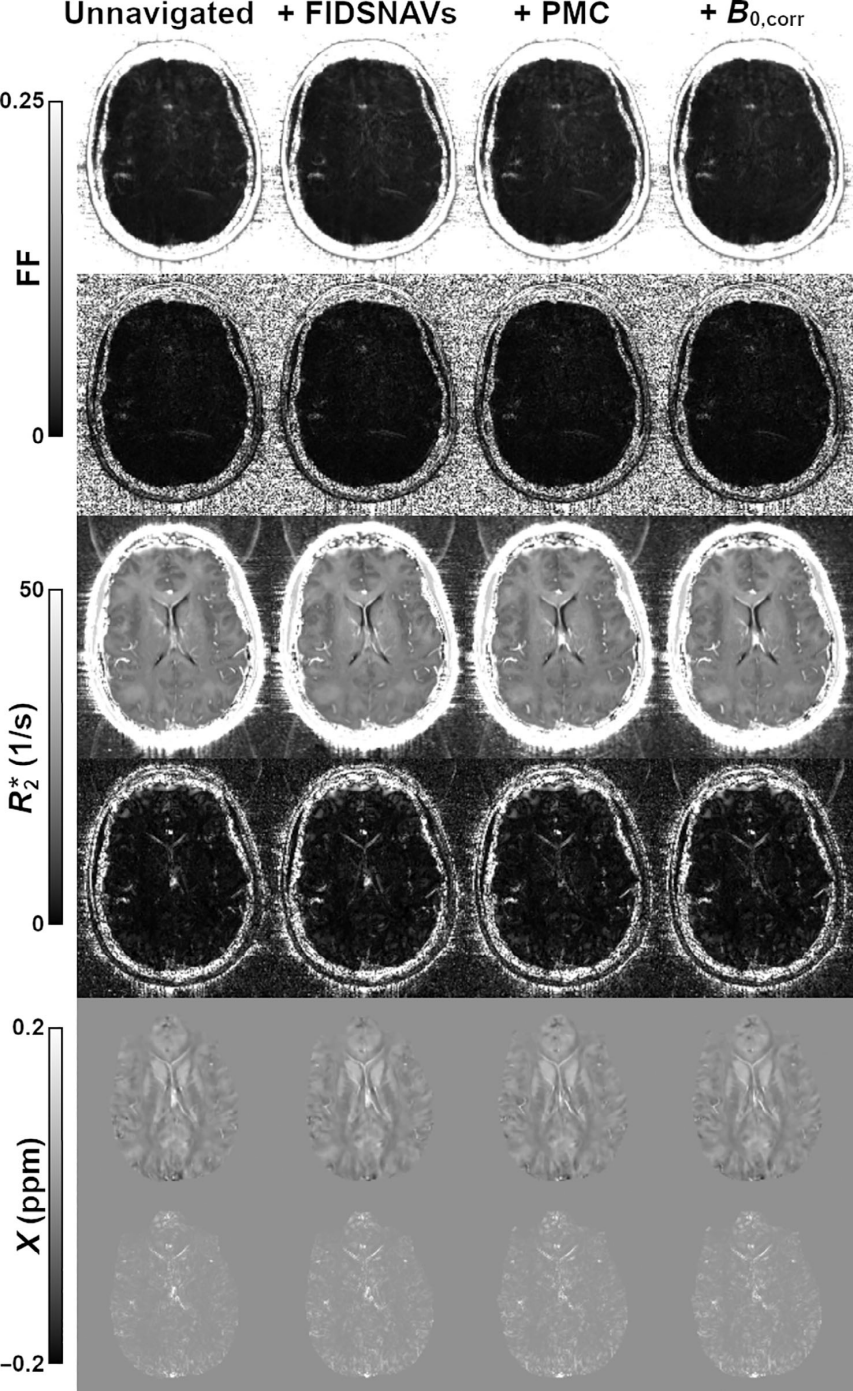
Sample maps of FF, $R_{2}^{*}$, and susceptibility from a case with no intentional motion verify that the proposed corrections do not introduce unwanted artifacts. Shown are the results obtained from scans from the unnavigated sequence, along with scans with navigator acquisition, PMC, and additional retrospective $B_{0}$ correction. Below each map is the difference relative to that derived from the reference scan (unnavigated). $B_{0,\text{corr}}$, retrospective field correction; FF, fat fraction; *X*, susceptibility.

Real‐time motion estimation from each FIDSNAV, including field correction, required a mean computation time of 21.9 ± 0.3 ms (maximum 28.6 ms), resulting in a total latency of under 40 ms when including margin time for the FOV update. With no intentional motion, FIDSNAVs with or without motion and field correction showed no visible impact on image quality. Figure 2 displays some of the sample quantitative maps, which also show no evidence of degradation. High FF was detected within arteries, and to a lesser extent within veins, but is likely artifactual. We attribute this to flow artifact because flow compensation was only performed for the first echo in the echo train.^36^ Supplementing this visual inspection, investigation of quantitative image quality metrics also found no clear evidence of deterioration relative to the unnavigated reference (results not shown).

Motion scores for each of the intentional motion cases are provided in Table 1. For the most part, similar degrees of motion were observed for scans with and without correction. It is important to note that this metric simply represents the distribution of motion states within a scan and not the motion pattern (e.g. slow drift vs. frequent readjustment). Therefore, it should not be taken as a predictor of image quality but rather an indicator of whether two scans with the same motion pattern/prompt have similar levels of motion.

**TABLE 1:** Summary of RMS motion scores with motion no correction, motion with PMC, and no motion (reference scan and additional repetition).

	RMS motion score (mm)			
			No motion	No motion
Case	Motion	+PMC	(Rep)	(Ref)
Case 1	2.3	1.6	1.2	0.6
Case 2	1.1	1.1	0.7	0.8
Case 3	2.5	1.7	0.7	0.9+
Case 4	2.9+	2.6+	0.4	0.7+
Case 5	4.4+	5.7+	0.7	1.4

*Note*: Cases with possible rotation outside the detectable range ±6° are denoted with a plus sign.

PMC, prospective motion correction.

**FIGURE 3:**
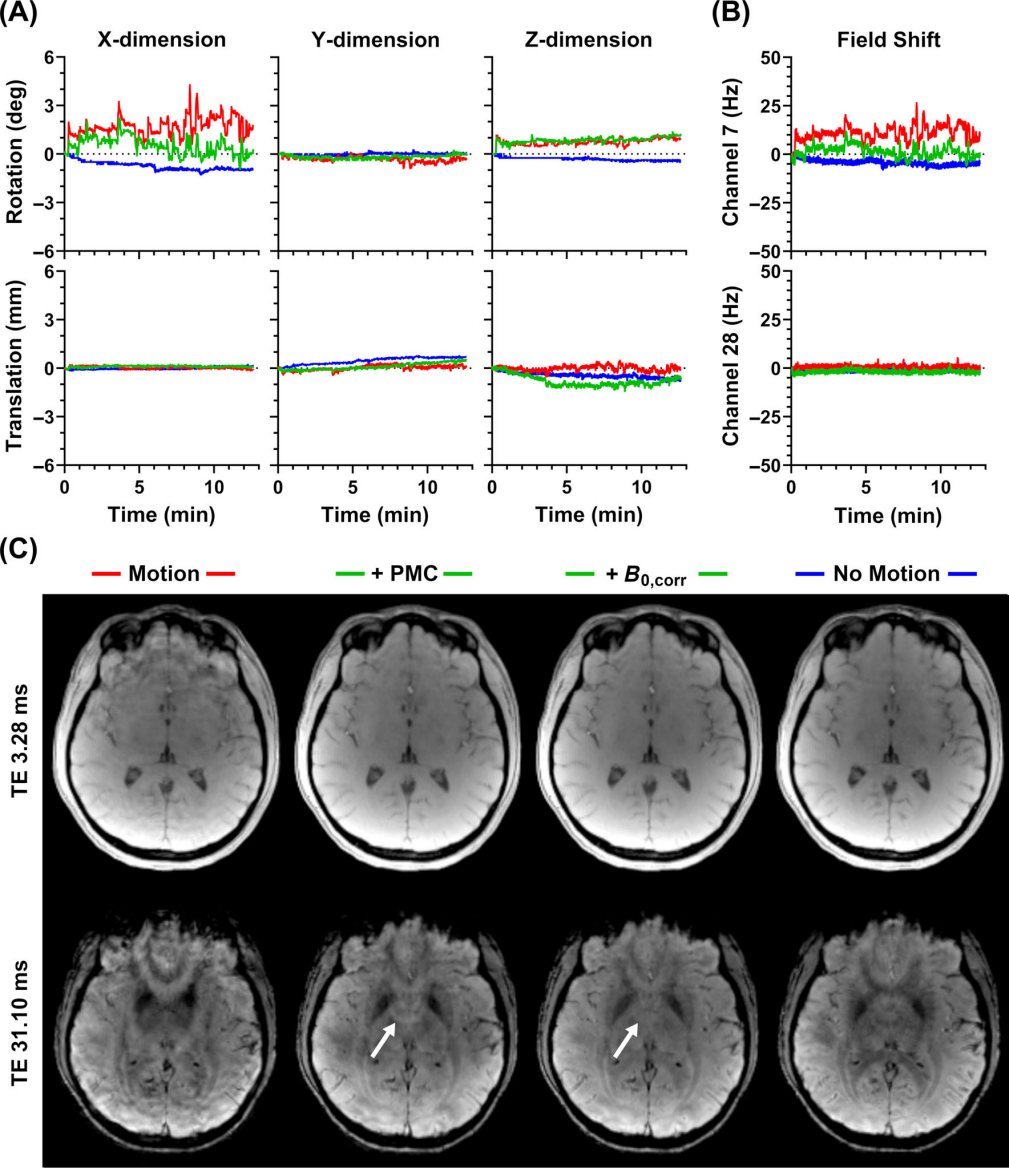
Measured (A) motion traces and (B) field shifts (shown for two sample channels) for a moderate motion case (case 2). (C) Sample magnitude images acquired with motion no correction, motion with PMC, additional retrospective field correction, and no motion reference (shown for echoes one and eight).

**FIGURE 4:**
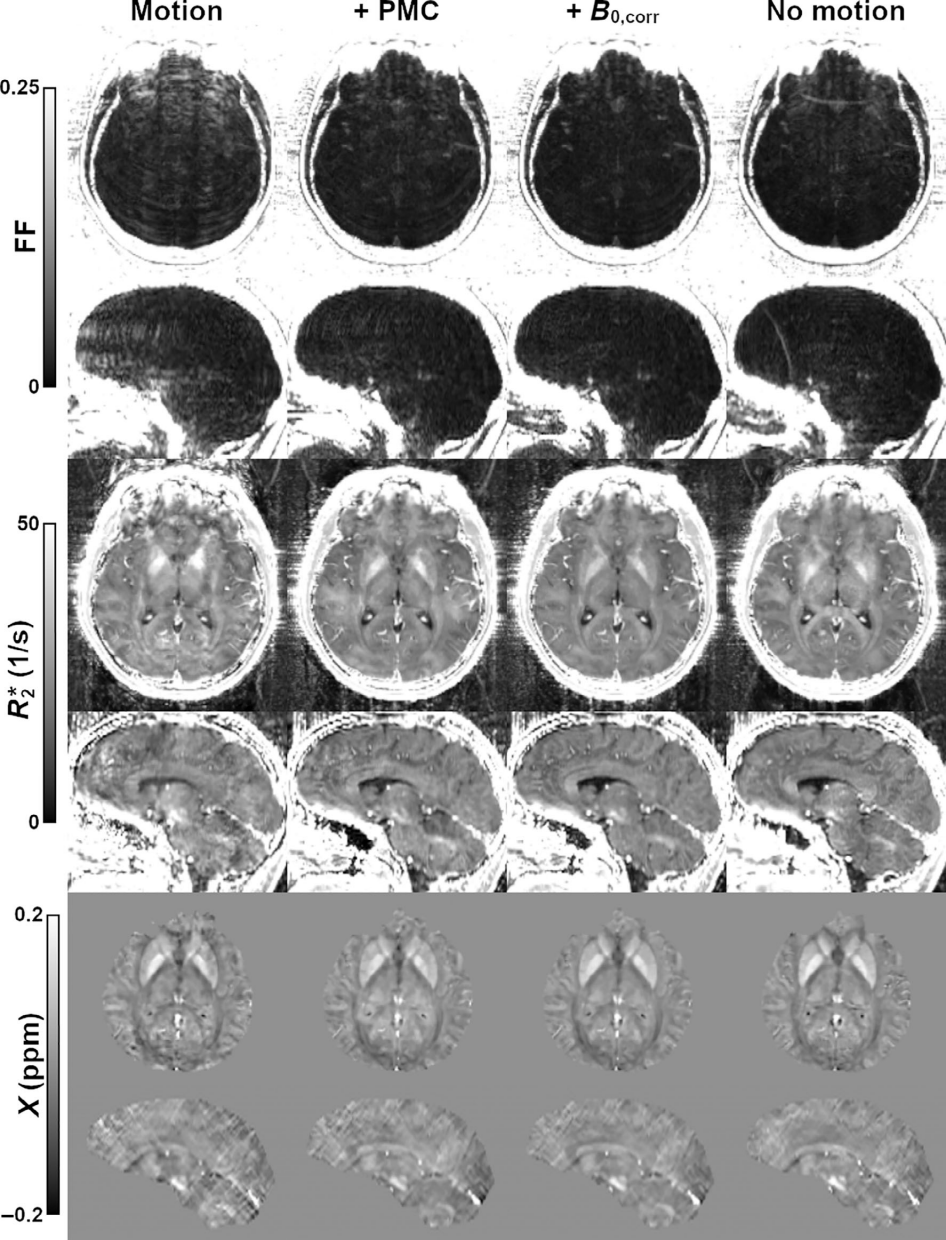
Axial and sagittal views of FF, $R_{2}^{*}$, and susceptibility for case 2 (moderate motion). Motion leads to clear overestimation of FF within the brain, largely reduced with PMC. Additional field correction improves, for example, homogeneity of $R_{2}^{*}$ within the corpus callosum.

Sample traces, field shifts for case 2 are given in Figures 3A,B, respectively. Although offset, the relative degree of motion and field shift throughout the corrected and uncorrected scans is comparable. Measured field shifts vary greatly depending on the channel; for reference, channel 7 was most sensitive to the frontal lobe, whereas channel 28 was located superior to the left parietal lobe. Corresponding magnitude images are given in Figure 3C, showing a moderate degree of artifacts. At the short TE, there is a clear reduction in artifacts with PMC alone, whereas additional improvement with field correction is minimal. This field correction becomes more valuable at the longer TE, where it appears to provide an overall improvement in signal homogeneity; for example, residual artifacts between the left and right globi pallidi are reduced. Corresponding quantitative maps are provided in Figure 4. Ringing leads to overestimation of FF measurements within the brain. This effect is largely removed with PMC alone, although additional, more minor improvement is visible with the field correction. Improvement using PMC is also clear in the resulting $R_{2}^{*}$ and susceptibility maps. The benefit of further field correction may be highlighted by the improvement in $R_{2}^{*}$ homogeneity within the sagittal view of the corpus callosum.

**FIGURE 5:**
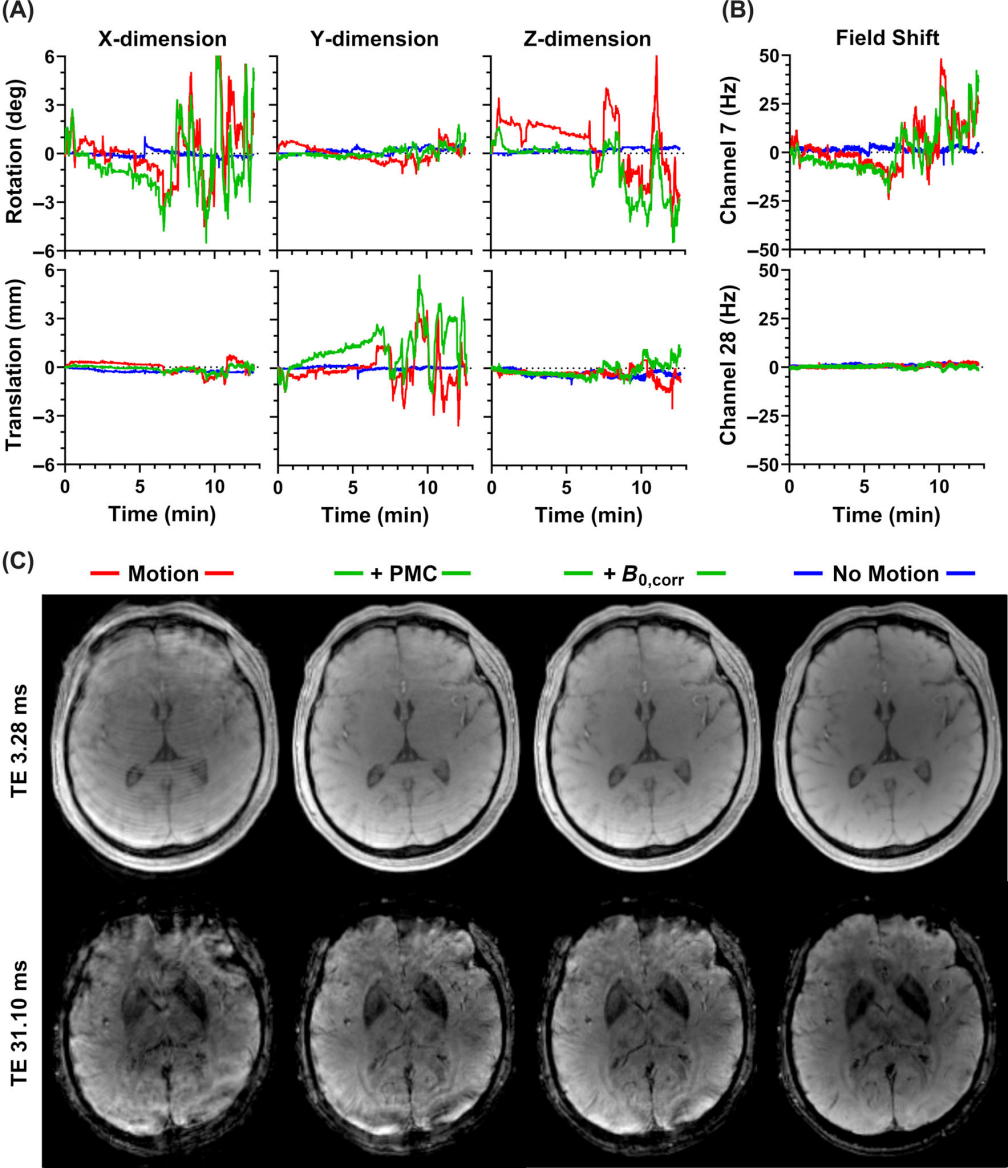
Measured (A) motion traces and (B) field shifts (shown for two sample channels) for a more severe motion case (case 5). (C) Sample magnitude images acquired with motion no correction, motion with PMC, additional retrospective field correction, and the no motion reference (shown for echoes one and eight).

**FIGURE 6:**
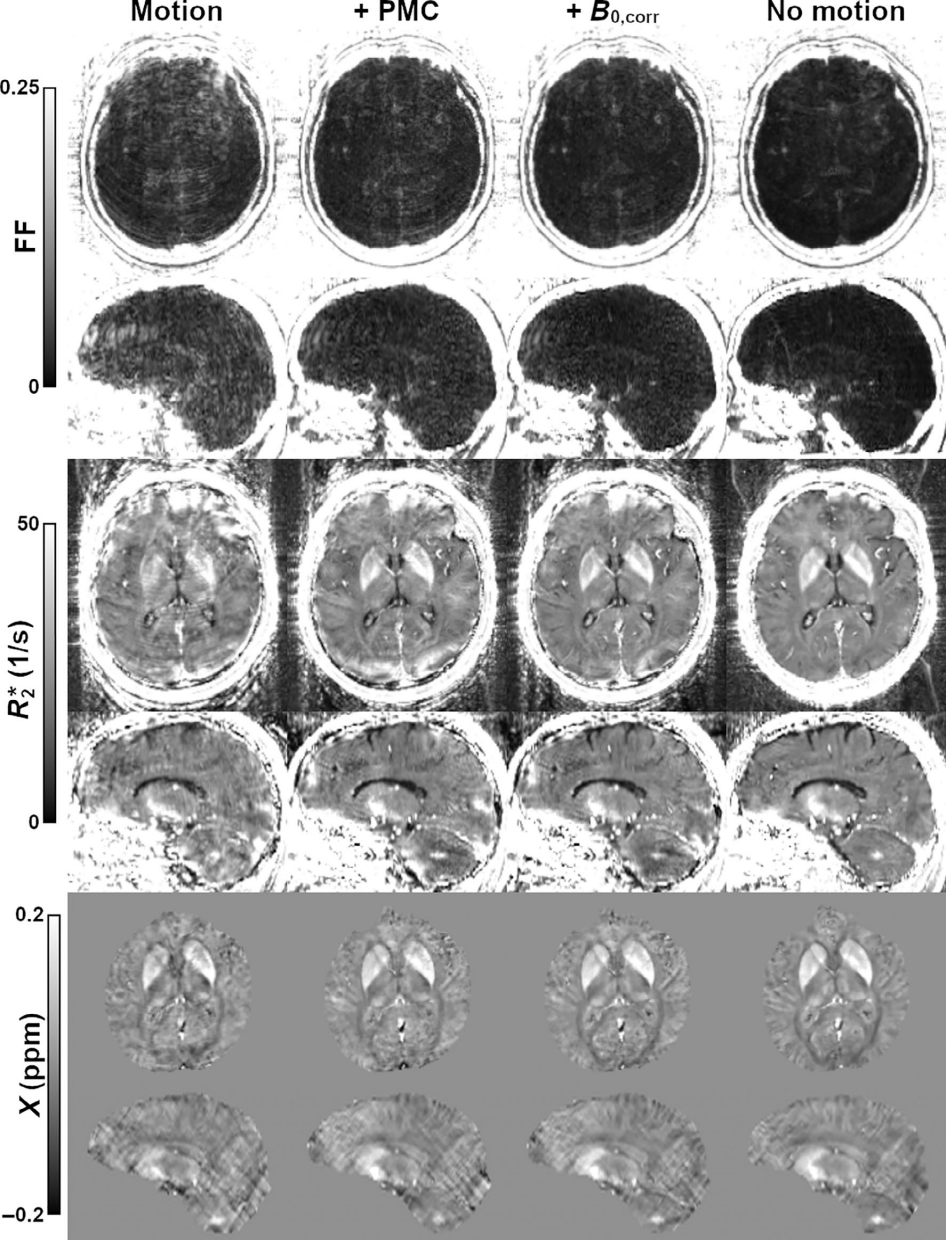
Axial and sagittal views of FF, $R_{2}^{*}$, and susceptibility for case 5 (severe motion). Improvement with PMC is clear; additional value of field correction can be seen in the right posterior of the axial brain views. Residual artifacts are more apparent in this case; see the cerebellum, posterior occipital lobe, and anterior frontal lobe.

Sample traces, field shifts for case 5 are given in Figures 5A,B, respectively. Comparable motions were observed in cases with and without PMC, again offset in some instances. Corresponding magnitude images are given in Figure 5C; the motion artifacts observed in this case were among the most severe observed across all participants. As before, PMC alone provides a notable improvement in image quality at the earliest TE; however, some residual ringing was observed in this case, and little improvement was achieved with further field correction. The effect of field correction is clearer at the later TE, largely correcting the signal void in the right posterior of the brain; although residual artifacts are also more apparent. Resulting quantitative maps are displayed in Figure 6. Improvement with PMC is clear in all cases. As was the case in Figure 5, the benefit of further field correction for $R_{2}^{*}$ and susceptibility mapping may be highlighted in the right posterior of the axial brain views. Residual artifacts are most apparent in the cerebellum, posterior occipital lobe, and anterior frontal lobe.

**FIGURE 7:**
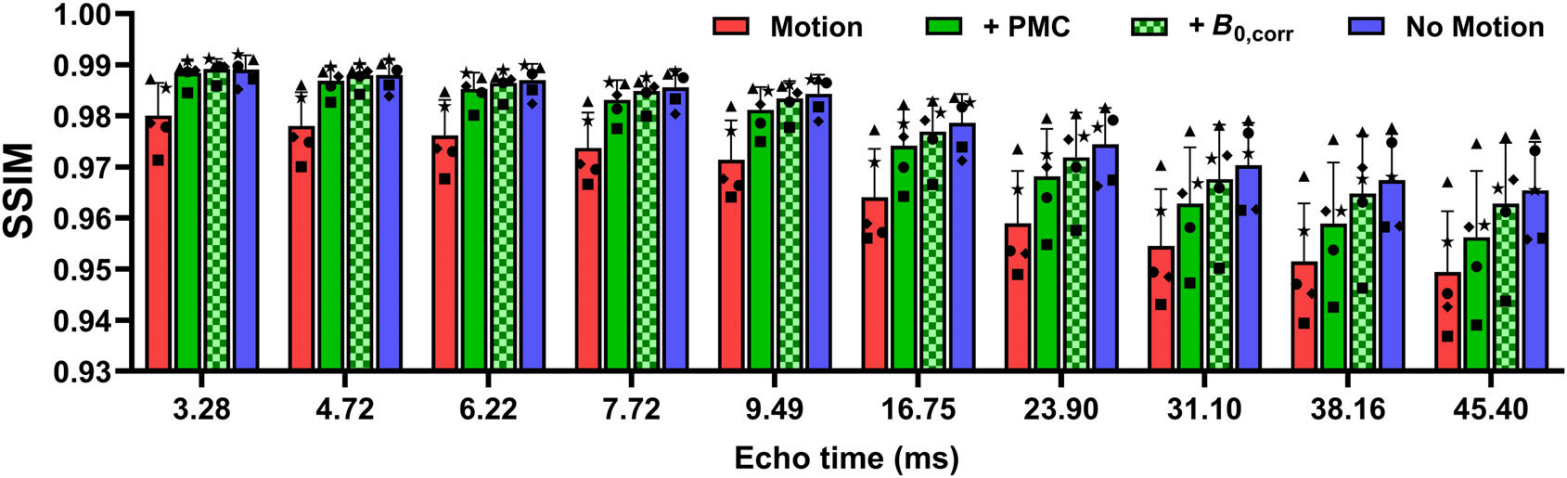
Magnitude image quality (quantified using SSIM) as a function of TE for all participants for cases of motion no correction, motion with PMC with and without additional retrospective field correction. Also shown are the results for a no motion repetition, providing a measure of error that can be attributed to interscan variability. For reference, cases 2 and 5 displayed in the preceding figures are labeled with stars and squares, respectively. SSIM, structural similarity.

Sample traces, field shifts, magnitude images, and quantitative maps for one additional case are provided in Figures S3 and S4 (minor artifacts). Full quantitative mapping results for the sample cases are also provided in Videos [Link], [Link]. An overview of echo‐wise image quality as quantified using structural similarity is provided in Figure 7. At the shortest TEs, PMC alone resulted in measures of error approaching that which can be attributed to interscan variability (i.e. error between two scans with no intentional motion). Additional field correction provided consistent but minimal improvement. For later TEs, improvement in image quality with additional field correction was much more apparent; however, residual error remains that cannot be entirely attributed to interscan variability. Similar trends were observed using NRMSE (provided in Figure S2), but values were found to be overly sensitive to interscan variations in low spatial‐frequency signal inhomogeneity (possibly caused by motion‐induced changes in coil sensitivity).

**FIGURE 8:**
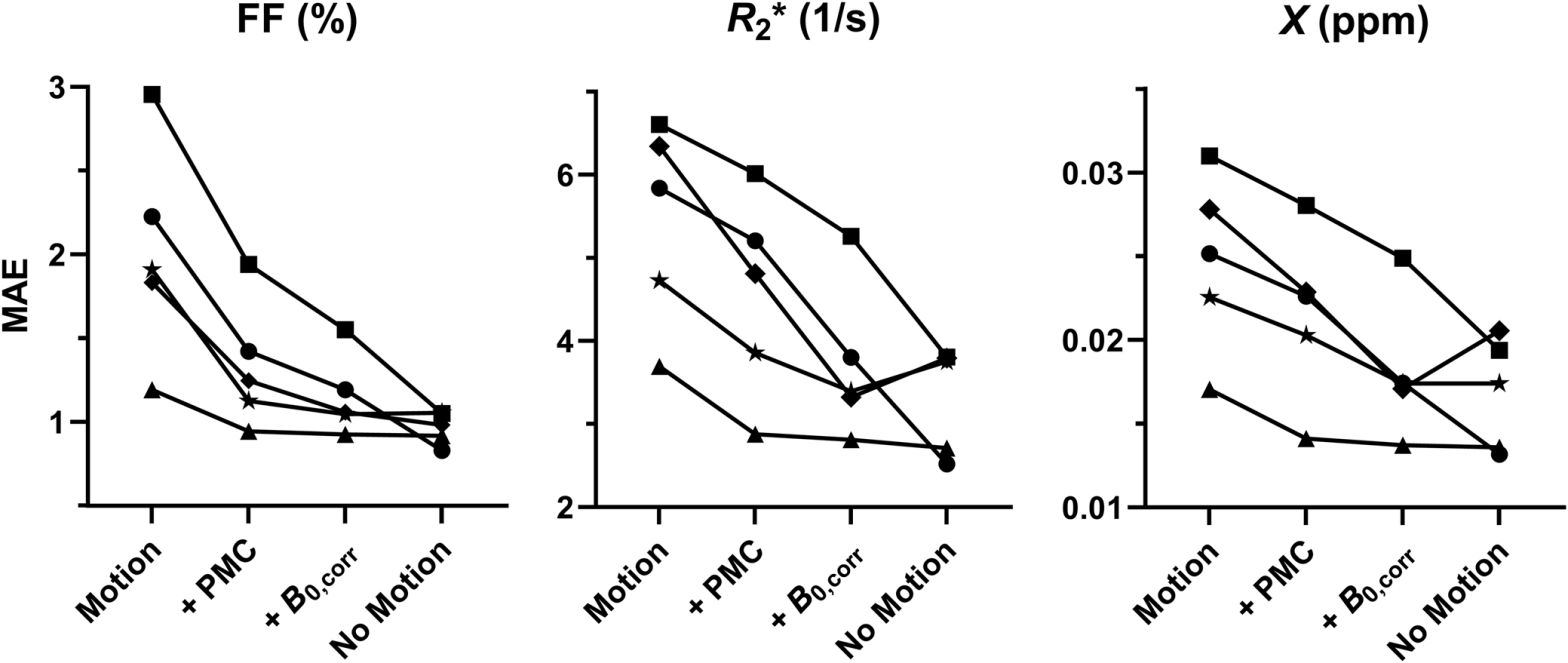
Brain‐wide mapping error (quantified using MAE) for FF, $R_{2}^{*}$, and susceptibility for all participants. For comparison, cases 2 and 5 are labeled with stars and squares, respectively. MAE, mean absolute error.

Errors in the corresponding quantitative maps are summarized in Figure 8. Maps of FF benefited from the largest improvement in image quality using PMC alone, although additional retrospective field correction did further reduce error in all cases. In the case of $R_{2}^{*}$ mapping and QSM, both motion and field correction provided a notable and consistent reduction in quantitative mapping error.

## 4 DISCUSSION

This work demonstrates a purely navigator‐based approach to PMC for $R_{2}^{*}$ mapping or QSM, further addressing motion‐induced field inhomogeneity. We show clear and consistent reduction of artifacts in the magnitude images and resulting quantitative maps. Given the long TEs typically used in measuring these properties, both PMC and retrospective $B_{0}$ correction were important for reduced quantitative mapping error. Residual artifacts were observed in the most severe motion case (case 5); possible improvements are discussed later.

Because FF maps were produced as part of the same postprocessing pipeline, we also demonstrate the benefit of motion and/or field correction for this application. In this case, PMC alone was often sufficient to remove the majority of motion artifacts; although consistent, the improvement with additional field correction was relatively minor. This was expected because FF was derived from data acquired early in the echo train (TEs < 10 ms), where phase inconsistencies accrued due to motion‐induced field inhomogeneity are still small. At the earliest TE (3.28 ms), image quality using PMC alone approached that which can be attributed to interscan variability (Figure 7). The good performance of PMC in this case is attributed to the frequency (1.96 Hz) and low latency (under 40 ms) of the FOV update, as well as the accuracy of motion estimates (section S1).

In fact, some prior works have investigated motion correction for $R_{2}^{*}$ mapping and/or QSM without considering the additional effects of motion‐induced field inhomogeneity.^16^, ^17^, ^23^ Good results may be achieved if the motion is very small, for example, within 2 mm^17^, ^23^ as seen in the results of case 1 (triangle markers in Figure 8, Figures S3 and S4). However, larger motions can limit the consistency of quantitative mapping using PMC alone, particularly for tissue properties such as $R_{2}^{*}$ which are more reliant on data acquired at longer TEs.^16^ As a result, more recent approaches to motion correction for $R_{2}^{*}$ mapping and/or QSM have opted to incorporate field correction.^21^, ^24^, ^28^ For example, VNAVs may be used for simultaneous motion and field map estimation;^25^, ^26^, ^27^ however, such approaches for $R_{2}^{*}$ mapping and/or QSM have been limited to retrospective motion correction,^24^, ^28^ which are proven to have worse performance compared to prospective approaches.^15^ PMC using optical tracking has been combined with FID navigators for motion and field correction of $R_{2}^{*}$ mapping,^21^ but a purely navigator‐based approach as demonstrated in this work may be more suitable for widespread implementation.

Although the FIDSNAV‐based approach to combined PMC and retrospective $B_{0}$ correction presented in this work is advantageous in that it requires no external hardware, it is not without its limitations. Residual quantitative mapping error was apparent in the most severe motion case (Figure 6). Performance may have been limited by rotations exceeding the detectable range of ±6°, the frequency of motion estimation (1.96 Hz, limited due to tradeoff with scan time, discussed later), and other factors not accounted for with PMC, including changes in receive coil sensitivity.^54^ It is also worth noting that QSM depends on orientation relative to $B_{0}$, which likely limits correction for larger rotations. However, the clear increase in residual artifacts at longer TEs (Figures 5 and 7) would indicate that inadequate field correction is the primary concern. Whereas this work implemented field correction on a channel‐wise basis to partially account for spatial variations in field fluctuation, each channel was still corrected for zeroth‐order field changes alone (section 2.5). Higher‐order field monitoring and prospective field correction could better account for the motion‐induced field inhomogeneity. In fact, the additional FID navigator could be used for higher order field monitoring if additional reference data is acquired,^55^, ^56^, ^57^ though processing time for prospective correction will have to be assessed. Real‐time shimming of higher‐order field changes may only be possible with specialized hardware.^58^ When performing $R_{2}^{*}$ mapping and/or QSM in motion‐prone populations, protocols making use of shorter TEs^59^ may also prove beneficial in limiting the effects of motion‐induced field inhomogeneity. For demonstration purposes, the large range of TEs in the protocol employed in this work was desirable to test the robustness of the technique.

Employing a protocol with shorter TEs would also enable shorter TRs, reducing the time needed to acquire the FIDSNAV baselines (important to limit motion during this period) and scan time increase associated with navigator acquisition throughout imaging. For this work, total scan time increase associated with navigation was considerable, approaching 30%, because the navigators had to be acquired fairly often (every eight k‐space lines) to maintain a reasonable motion estimation frequency (1.96 Hz). Alternatively, it may be possible to reduce acquisition time of the navigator acquisition itself. For this work, SNAVs were acquired as two hemispheres to prevent linear phase ramps caused by $B_{0}$ inhomogeneity; however, this may not be required with the additional FID‐based field correction (reducing acquisition time by a factor of two). It is also possible that good results could be achieved with a lower update frequency;^32^ ideally, navigator acquisition could be optimized on a case‐by‐case basis with FID‐based triggering.^60^, ^61^

If implemented together with prospective update of the navigator trajectory, optimization of navigator frequency to limit the amount of motion between successive acquisitions would reduce the requirements of the baseline acquisition, which must currently cover the entire range of motion expected throughout the scan (±6°). In fact, for very small motions (tenths of a degree) the motion estimation process can be simplified to a linear modeling problem with greatly reduced calibration requirements.^2^, ^3^ However, maintaining this linear domain for complex motion trajectories (e.g. case 5) would require a higher navigator frequency. For alternative protocols containing more dead time, in which case navigator acquisition may be performed at high frequency without scan time penalty, a linear modeling approach with little or no reference data prerequisites may be preferable.^2^, ^3^ In this work, the density of the imaging echo train prompted navigator acquisition with a separate excitation; therefore, the lookup table approach was desirable because it enabled a more flexible tradeoff between navigator acquisition frequency and scan time.

Lastly, it is also important to note several factors limiting the comparisons performed in this work. For one, the nature of PMC precludes direct comparison with the uncorrected counterpart, which must instead be obtained in a separate acquisition. For in vivo testing with human volunteers, this motion cannot be exactly replicated. We attempted to control for this by providing participants with a visual indicator, although many cases still demonstrated some degree of inconsistency in motion scale (Table 1). Secondly, the no motion scans are not necessarily free of motion. In fact, one of the no motion scans for case 1 was reacquired due to motion artifacts detected during the scanning session, and other cases may have experienced more minor motion or field inhomogeneity artifacts due to gradual drift, breathing, or unintentional spurious motion that went undetected. Therefore, the measures of error attributed to interscan variability in Figures 7 and 8 are likely overestimated. We also note that, as highlighted in Figure S2, the quantitative measures of image quality used to evaluate the magnitude images in this work may not provide the best indicator of image quality;^62^ although error in the quantitative maps can be taken as a fairly unbiased metric. For this work, quantitative assessment was limited to the brain as a whole; other regions were excluded to reduce errors related to nonrigid deformation (occurring mainly near the ears and nose). The effect of correction within smaller regions of interest could not be assessed with the small sample size presented here. Future work will assess the effects of motion bias and correction more completely using region of interest analysis at a larger scale.

## 5 CONCLUSION

This work presents a purely navigator‐based approach to PMC of $R_{2}^{*}$ and susceptibility mapping. Implementing FIDSNAVs for combined PMC and retrospective field correction consistently reduced quantitative mapping error in the presence of motion. Potential applications include brain iron quantification in the study of movement disorders.

## FUNDING INFORMATION

Funding was provided by the Natural Sciences and Engineering Research Council of Canada (NSERC), grant RGPIN 05835‐2016; and the Canadian Institutes of Health Research (CIHR), grant PJT159665.

## CONFLICTS OF INTEREST

Omer Oran is an employee of Siemens Healthcare Limited.

## Supporting information


**Figure S1.** Plots of expected (blue) and measured (red) trajectories of rotation and displacement from phantom experiments with FID‐based field correction of SNAV data. The same data is shown on the right in the form of calibration plots.
**Figure S2.** Magnitude image quality (quantified using NRMSE) as a function of echo time for all participants for cases of motion no correction, motion with PMC with and without additional retrospective field correction. Also shown are the results for a no motion repetition, meant to provide a reasonable measure of error which can be attributed to interscan variability. However, at least one such case (Case 3, denoted by the diamond symbols) demonstrated inflated error with no clear motion artifacts due to interscan variations in low‐spatial‐frequency signal inhomogeneity (attributed to motion‐induced coil‐sensitivity fluctuations relative to the reference acquisition). NRMSE, normalized RMSE.
**Figure S3.** Measured (A) motion traces and (B) field shifts (shown for two sample channels) for a minor motion case (Case 1). (C) Sample magnitude images acquired with motion no correction, motion with PMC, additional retrospective field correction, and the no motion reference (shown for echoes one and eight).
**Figure S4.** Axial and sagittal views of fat fraction, $R_{2}^{*}$, and susceptibility for Case 1 (minor motion).

**Video S1.** All axial slices of the sample quantitative maps shown in Figure 4 (Case 2) with skull stripping to preserve anonymity.

**Video S2.** All axial slices of the sample quantitative maps shown in Figure 6 (Case 5) with skull stripping to preserve anonymity.

**Video S3.** All axial slices of the sample quantitative maps shown in Figure S4 (Case 1) with skull stripping to preserve anonymity.

## Data Availability

The custom navigated sequence and online reconstruction pipeline for prospective motion correction is available within the Siemens C2P exchange.

## References

[1] Reeder SB , McKenzie CA , Pineda AR , et al. Water‐fat separation with IDEAL gradient‐echo imaging. J Magn Reson Imaging. 2007;25:644‐652. 17326087 10.1002/jmri.20831

[2] Fernández‐Seara MA , Wehrli FW . Postprocessing technique to correct for background gradients in image‐based R2* measurements. Magn Reson Med. 2000;44:358‐366. 10975885 10.1002/1522-2594(200009)44:3<358::aid-mrm3>3.0.co;2-i

[3] Liu J , Liu T , de Rochefort L , et al. Morphology enabled dipole inversion for quantitative susceptibility mapping using structural consistency between the magnitude image and the susceptibility map. Neuroimage 2012;59:2560–2568. 21925276 10.1016/j.neuroimage.2011.08.082 PMC3254812

[4] Martin WR , Wieler M , Gee M . Midbrain iron content in early Parkinson disease. Neurology. 2008;70:1411‐1417. 18172063 10.1212/01.wnl.0000286384.31050.b5

[5] Lotfipour AK , Wharton S , Schwarz ST , et al. High resolution magnetic susceptibility mapping of the substantia nigra in Parkinson's disease. J Magn Reson Imaging 2012;35:48–55. 21987471 10.1002/jmri.22752

[6] Alushaj E , Handfield‐Jones N , Kuurstra A , et al. Increased iron in the substantia nigra pars compacta identifies patients with early Parkinson's disease: a 3T and 7T MRI study. Neuroimage Clin 2024;41:103577. 38377722 10.1016/j.nicl.2024.103577 PMC10944193

[7] Sharma S , Sethi SK , Reese D , et al. Brain iron deposition and movement disorders in hereditary haemochromatosis without liver failure: a cross‐sectional study. Eur J Neurol 2022;29:1417–1426. 34989476 10.1111/ene.15242

[8] Andre JB , Bresnahan BW , Mossa‐Basha M , et al. Toward quantifying the prevalence, severity, and cost associated with patient motion during clinical MR examinations. J Am Coll Radiol 2015;12:689–695. 25963225 10.1016/j.jacr.2015.03.007

[9] Satterthwaite TD , Wolf DH , Loughead J , et al. Impact of in‐scanner head motion on multiple measures of functional connectivity: relevance for studies of neurodevelopment in youth. Neuroimage 2012;60:623–632. 22233733 10.1016/j.neuroimage.2011.12.063 PMC3746318

[10] Power JD , Barnes KA , Snyder AZ , Schlaggar BL , Petersen SE . Spurious but systematic correlations in functional connectivity MRI networks arise from subject motion. Neuroimage. 2012;59:2142‐2154. 22019881 10.1016/j.neuroimage.2011.10.018 PMC3254728

[11] Kim JH , Asis‐Cruz JD , Kapse K , Limperopoulos C . Systematic evaluation of head motion on resting‐state functional connectivity MRI in the neonate. Hum Brain Mapp 2023;44:1934‐1948. 36576333 10.1002/hbm.26183 PMC9980896

[12] Reuter M , Tisdall MD , Qureshi A , Buckner RL , van der Kouwe AJW , Fischl B . Head motion during MRI acquisition reduces gray matter volume and thickness estimates. Neuroimage 2015;107:107‐115. 25498430 10.1016/j.neuroimage.2014.12.006 PMC4300248

[13] Tisdall MD , Reuter M , Qureshi A , Buckner RL , Fischl B , van der Kouwe AJW . Prospective motion correction with volumetric navigators (vNavs) reduces the bias and variance in brain morphometry induced by subject motion. Neuroimage 2016;127:11‐22. 26654788 10.1016/j.neuroimage.2015.11.054 PMC4754677

[14] Maclaren J , Herbst M , Speck O , Zaitsev M . Prospective motion correction in brain imaging: a review. Magn Reson Med 2013;69:621‐636. 22570274 10.1002/mrm.24314

[15] Slipsager JM , Glimberg SL , Højgaard L , et al. Comparison of prospective and retrospective motion correction in 3D‐encoded neuroanatomical MRI. Magn Reson Med 2022;87:629–645. 34490929 10.1002/mrm.28991 PMC8635810

[16] Callaghan MF , Josephs O , Herbst M , Zaitsev M , Todd N , Weiskopf N . An evaluation of prospective motion correction (PMC) for high resolution quantitative MRI. Front Neurosci 2015;9:97. 25859178 10.3389/fnins.2015.00097 PMC4373264

[17] Mattern H , Sciarra A , Lüsebrink F , Acosta‐Cabronero J , Speck O . Prospective motion correction improves high‐resolution quantitative susceptibility mapping at 7T. Magn Reson Med 2019;81:1605‐1619. 30298692 10.1002/mrm.27509

[18] Versluis MJ , Sutton BP , de Bruin PW , Börnert P , Webb AG , van Osch MJ . Retrospective image correction in the presence of nonlinear temporal magnetic field changes using multichannel navigator echoes. Magn Reson Med 2012;68:1836‐1845. 22362637 10.1002/mrm.24202

[19] Wezel J , Boer VO , van der Velden TA , et al. A comparison of navigators, snap‐shot field monitoring, and probe‐based field model training for correcting B0‐induced artifacts in T2*‐weighted images at 7T. Magn Reson Med 2017;78:1373–1382. 27859614 10.1002/mrm.26524

[20] Loktyushin A , Ehses P , Schölkopf B , Scheffler K . Autofocusing‐based phase correction. Magn Reson Med 2018;80:958‐968. 29352498 10.1002/mrm.27092

[21] Vaculčiaková L , Podranski K , Edwards LJ , et al. Combining navigator and optical prospective motion correction for high‐quality 500 um resolution quantitative multi‐parameter mapping at 7T. Magn Reson Med 2022;88:787–801. 35405027 10.1002/mrm.29253

[22] White N , Roddey C , Shankaranarayanan A , et al. PROMO: real‐time prospective motion correction in MRI using image‐based tracking. Magn Reson Med 2010;63:91–105. 20027635 10.1002/mrm.22176 PMC2892665

[23] Bazin PL , Nijsse HE , van der Zwaag W , et al. Sharpness in motion corrected quantitative imaging at 7T. Neuroimage 2020;222:117227. 32781231 10.1016/j.neuroimage.2020.117227

[24] Dong Z , Wang F , Setsompop K . Motion‐corrected 3D‐EPTI with efficient 4D navigator acquisition for fast and robust whole‐brain quantitative imaging. Magn Reson Med 2022;88:1112‐1125. 35481604 10.1002/mrm.29277 PMC9246907

[25] Hess AT , Tisdall MD , Andronesi OC , Meintjes EM , van der Kouwe AJW . Real‐time motion and B0 corrected single voxel spectroscopy using volumetric navigators. Magn Reson Med 2011;66:314‐323. 21381101 10.1002/mrm.22805 PMC3123687

[26] Alhamud A , Taylor PA , van der Kouwe AJW , Meintjes EM . Real‐time measurement and correction of both B0 changes and subject motion in diffusion tensor imaging using a double volumetric navigated (DvNav) sequence. Neuroimage 2016;126:60‐71. 26584865 10.1016/j.neuroimage.2015.11.022 PMC4733594

[27] Simegn GL , van der Kouwe AJW , Robertson FC , Meintjes EM , Alhamud A . Real‐time simultaneous shim and motion measurement and correction in glycoCEST MRI using double volumetric navigators (DvNavs). Magn Reson Med 2019;81:2600‐2613. 30506877 10.1002/mrm.27597 PMC7251754

[28] van Gelderen P , Li X , de Zwart JA , et al. Effect of motion, cortical orientation and spatial resolution on quantitative imaging of cortical R2* and magnetic susceptibility at 0.3 mm in‐plane resolution at 7 T. Neuroimage 2023;27:119992. 10.1016/j.neuroimage.2023.119992 PMC10278242 36858332

[29] van der Kouwe AJW , Benner T , Dale AM . Real‐time rigid body motion correction and shimming using cloverleaf navigators. Magn Reson Med 2006;56:1019‐1032. 17029223 10.1002/mrm.21038

[30] Ulrich T , Riedel M , Pruessmann KP . Servo navigators: linear regression and feedback control for rigid‐body motion correction. Magn Reson Med 2024;91:1876‐1892. 38234052 10.1002/mrm.29967

[31] Riedel M , Ulrich T , Pruessmann KP . Run‐time motion and first‐order shim control by expanded servo navigation. Magn Reson Med 2025;93:166‐182. 39188123 10.1002/mrm.30262

[32] Hewlett M , Oran O , Liu J , Drangova M . Prospective motion correction for brain MRI using spherical navigators. Magn Reson Med 2024;91:1528‐1540. 38174443 10.1002/mrm.29961

[33] Johnson PM , Liu J , Drangova M . Simultaneous motion and B0 correction using FID‐SNAVs. Proceedings of the 25th Annual Meeting of the ISMRM. International Society for Magnetic Resonance in Medicine; 2017 p. 3948.

[34] Hewlett M , Liu J , Drangova M . Combined motion and B0 correction of susceptibility weighted imaging with jointly acquired FID and spherical navigators. *Proceedings of the Joint ISMRM & ISMRT Annual Meeting, Toronto, Ontario, Canada;* 2023. p. 1822.

[35] Hewlett M , Oran O , Liu J , Drangova M . Motion and B0 correction for multiparametric mapping with jointly acquired FID and spherical navigators. Proceedings of the Joint ISMRM & ISMRT Annual Meeting. International Society for Magnetic Resonance in Medicine; 2024 p. 2662.

[36] Liu J , Christiansen SD , Drangova M . Single multi‐echo GRE acquisition with short and long echo spacing for simultaneous quantitative mapping of fat fraction, B0 inhomogeneity, and susceptibility. Neuroimage. 2018;172:703‐717. 29448076 10.1016/j.neuroimage.2018.02.012

[37] Tisdall MD , Bhat H , Heberlein K , van der Kouwe AJW . Prospective head motion correction in 3D FLASH using EPI‐based volumetric navigators (vNavs). Proceedings of the 22nd Annual Meeting of the ISMRM. International Society for Magnetic Resonance in Medicine; 2014 p. 0882.

[38] Johnson PM , Liu J , Wade T , Tavallaei MA , Drangova M . Retrospective 3D motion correction using spherical navigator echoes. Magn Reson Imaging 2016;34:1274‐1282. 27451402 10.1016/j.mri.2016.06.006

[39] Hess AT , Alfaro‐Almagro F , Andersson JL , Smith SM . Head movement in UK Biobank, analysis of 42,874 fMRI motion logs. Proceedings of the ISMRM Workshop on Motion Detection & Correction. International Society for Magnetic Resonance in Medicine; 2022.

[40] Pfeuffer J , van de Moortele PF , Ugurbil K , Hu X , Glover GH . Correction of physiologically induced global off‐resonance effects in dynamic echo‐planar and spiral functional imaging. Magn Reson Med 2002;47:344‐353. 11810679 10.1002/mrm.10065

[41] Liu J , Drangova M . Phase‐unwrapping algorithm for translation extraction from spherical navigator echoes. Magn Reson Med 2010;63:510‐516. 19918896 10.1002/mrm.22198

[42] Welch EB , Manduca A , Grimm RC , Ward HA , Jack CR . Spherical navigator echoes for full 3D rigid body motion measurement in MRI. Magn Reson Med 2002;47:32‐41. 11754440 10.1002/mrm.10012

[43] Berglund J . MotionParadigm. gitlab.com/johan.berglund/MotionParadigm. Accessed February 3, 2022.

[44] Skare S , Brain MRI . Motion Data Base. brainmrimotion.org. Accessed June 12, 2023.

[45] Tisdall MD , Hess AT , Reuter M , Meintjes EM , Fischl B , van der Kouwe AJW . Volumetric navigators for prospective motion correction and selective reacquisition in neuroanatomical MRI. Magn Reson Med 2012;68:389‐399. 22213578 10.1002/mrm.23228 PMC3320676

[46] Chiew M . GRAPPA Reconstruction Tools. github.com/mchiew/grappa‐tools. Accessed September 30, 2022.

[47] Griswold MA , Jakob PM , Heidemann RM , et al. Generalized autocalibrating partially parallel acquisitions (GRAPPA). Magn Reson Med 2002;47:1202–1210. 12111967 10.1002/mrm.10171

[48] Uecker M , Lai P , Murphy MJ , et al. ESPIRiT — an eigenvalue approach to autocalibrating parallel MRI: where SENSE meets GRAPPA. Magn Reson Med 2014;71:990–1001. 23649942 10.1002/mrm.24751 PMC4142121

[49] Uecker M , Ong F , Tamir JI , et al. Berkeley advanced reconstruction toolbox. Proceedings of the 23rd Annual Meeting of the ISMRM. International Society for Magnetic Resonance in Medicine; 2015 p. 2486.

[50] Liu J , Drangova M . Method for B0 off‐resonance mapping by non‐iterative correction of phase‐errors (B0‐NICE). Magn Reson Med 2015;74:1177‐1188. 25351504 10.1002/mrm.25497

[51] Yu H , Shimakawa A , McKenzie CA , Brodsky E , Brittain JH , Reeder SB . Multiecho water‐fat separation and simultaneous R2* estimation with multifrequency fat spectrum modeling. Magn Reson Med 2008;60:1122‐1134. 18956464 10.1002/mrm.21737 PMC3070175

[52] Liu T , Wisnieff C , Lou M , Chen W , Spincemaille P , Wang Y . Nonlinear formulation of the magnetic field to source relationship for robust quantitative susceptibility mapping. Magn Reson Med 2013;69:467‐476. 22488774 10.1002/mrm.24272

[53] Zhou D , Liu T , Spincemaille P , Wang Y . Background field removal by solving the Laplacian boundary value problem. NMR Biomed 2013;27:312‐319. 10.1002/nbm.3064 24395595

[54] Rodriguez EP , Moser P , Auno S , et al. Real‐time motion and retrospective coil sensitivity correction for CEST using volumetric navigators (vNavs) at 7T. Magn Reson Med 2021;85:1909‐1923. 33165952 10.1002/mrm.28555 PMC7839562

[55] Utkur M , Wallace TE , Kober T , et al. Dynamic field correction for improved susceptibility weighted imaging with FID‐navigated 3D EPI. Proceedings of the Joint ISMRM & ISMRT Annual Meeting. International Society for Magnetic Resonance in Medicine; 2023 p. 1155.

[56] Salifu M , Noll D , Haskell M . Spatiotemporal B correction in Oscillating Steady State Imaging (OSSI) fMRI using Free Induction Decay Navigators (FIDnavs). Proceedings of the Joint ISMRM & ISMRT Annual Meeting. International Society for Magnetic Resonance in Medicine; 2023 p. 3420.

[57] Wallace TE , Kober T , Stockmann JP , Polimeni JR , K.Warfield S , Afacan O . Real‐time shimming with FID navigators. Magn Reson Med 2022;88:2548‐2563. 36093989 10.1002/mrm.29421 PMC9529812

[58] Andronesi OC , Frost R , Arango NS , et al. Real‐time motion correction and multicoil shim array B0 update for whole‐brain MR spectroscopic imaging. Proceedings of the Joint ISMRM & ISMRT Annual Meeting. International Society for Magnetic Resonance in Medicine; 2024 p. 0393. PMC13095162 42016486

[59] Chen Y , Liu S , Wang Y , Kang Y , Haacke EM . STrategically Acquired Gradient Echo (STAGE) imaging, part I: creating enhanced T1 contrast and standardized susceptibility weighted imaging and quantitative susceptibility mapping. Magn Reson Imaging 2018;46:130‐139. 29056394 10.1016/j.mri.2017.10.005

[60] Kober T , Marques JP , Gruetter R , Krueger G . Head motion detection using FID navigators. Magn Reson Med 2011;66:135‐143. 21337424 10.1002/mrm.22797

[61] Waszak M , Falkovskiy P , Hilbert T , et al. Prospective head motion correction using FID‐guided on‐demand image navigators. Magn Reson Med 2017;78:193–203. 27529516 10.1002/mrm.26364

[62] Mason A , Rioux J , Clarke SE , et al. Comparison of objective image quality metrics to expert Radiologists' scoring of diagnostic quality of MR images. IEEE Trans Med Imaging 2020;39:1064–1072. 31535985 10.1109/TMI.2019.2930338

