# Supplementary material for: Prospective motion correction for R2* and susceptibility mapping using spherical navigators
Source: Magn Reson Med. 2024 Dec 3;93(4):1642–56. doi: 10.1002/mrm.30385 (PMC11782710; doi:10.1002/mrm.30385)
Supplement: Supplementary file 1 — Figure S1. Plots of expected (blue) and measured (red) trajectories of rotation and displacement from phantom experiments with FID‐based field correction of SNAV data. The same data is shown on the right in the form of calibration plots. Figure S2. Magnitude image quality (quantified using NRMSE) as a function of echo time for all participants for cases of motion no correction, motion with PMC with and without additional retrospective field correction. Also shown are the results for a no motion repetition, meant to provide a reasonable measure of error which can be attributed to interscan variability. However, at least one such case (Case 3, denoted by the diamond symbols) demonstrated inflated error with no clear motion artifacts due to interscan variations in low‐spatial‐frequency signal inhomogeneity (attributed to motion‐induced coil‐sensitivity fluctuations relative to the reference acquisition). NRMSE, normalized RMSE. Figure S3. Measured (A) motion traces and (B) field shifts (shown for two sample channels) for a minor motion case (Case 1). (C) Sample magnitude images acquired with motion no correction, motion with PMC, additional retrospective field correction, and the no motion reference (shown for echoes one and eight). Figure S4. Axial and sagittal views of fat fraction, R2*, and susceptibility for Case 1 (minor motion). [file MRM-93-1642-s004.pdf]

S1. Phantom validation of motion tracking

A previous work has demonstrated the accuracy of SNAV-based motion estimation to be well within the sub-degree, millimeter range (31). However, given this may be influenced by acquisition parameters as well as the additional field correction, motion estimation accuracy was reassessed using a pineapple phantom (within the range ±6 deg, ±6 mm, based on expected values (38)). Sinusoidal rotations, performed sequentially about each axis, were simulated through update to the scanner FOV (62), while sinusoidal translations were produced using a 1D linear motion stage (63). The majority of this translation was expected to occur along the z-dimension; however, to obviate the need for calibration with the scanner's coordinate system, translation accuracy was measured in the form of total displacement. In both cases, navigators were interleaved with imaging acquisition (matched to that described in Section 2.3 Scan protocol) to account for all biases which may be present in the true implementation due to mismatched eddy current effects or other.

This phantom study of motion estimation accuracy showed very good agreement with simulated values (Supporting Information Figure S1), with mean absolute error in rotation estimation of 0.032 ± 0.056 deg (maximum error 0.36 deg). Similar results were achieved without FID correction as rotation estimation is based on magnitude signal (results not shown). Absolute error in displacement estimates averaged 0.23 ± 0.14 mm (maximum error 0.79 mm), resulting from slight bias in z-translation (around -0.10 mm), gradual negative drift in y-translation (-0.3 mm by end of scan), and slight underestimation for large displacements, potentially exaggerated by stage bending (31). Small oscillations in rotation were observed with oscillations in displacement (maximum error 0.25 deg). Given that SNAV acquisition is completed within 10 ms of RF excitation, FID correction did not have a large impact on motion estimates; although, performing estimation without FID correction did lead to overestimation of displacement values (maximum error 0.85 mm).

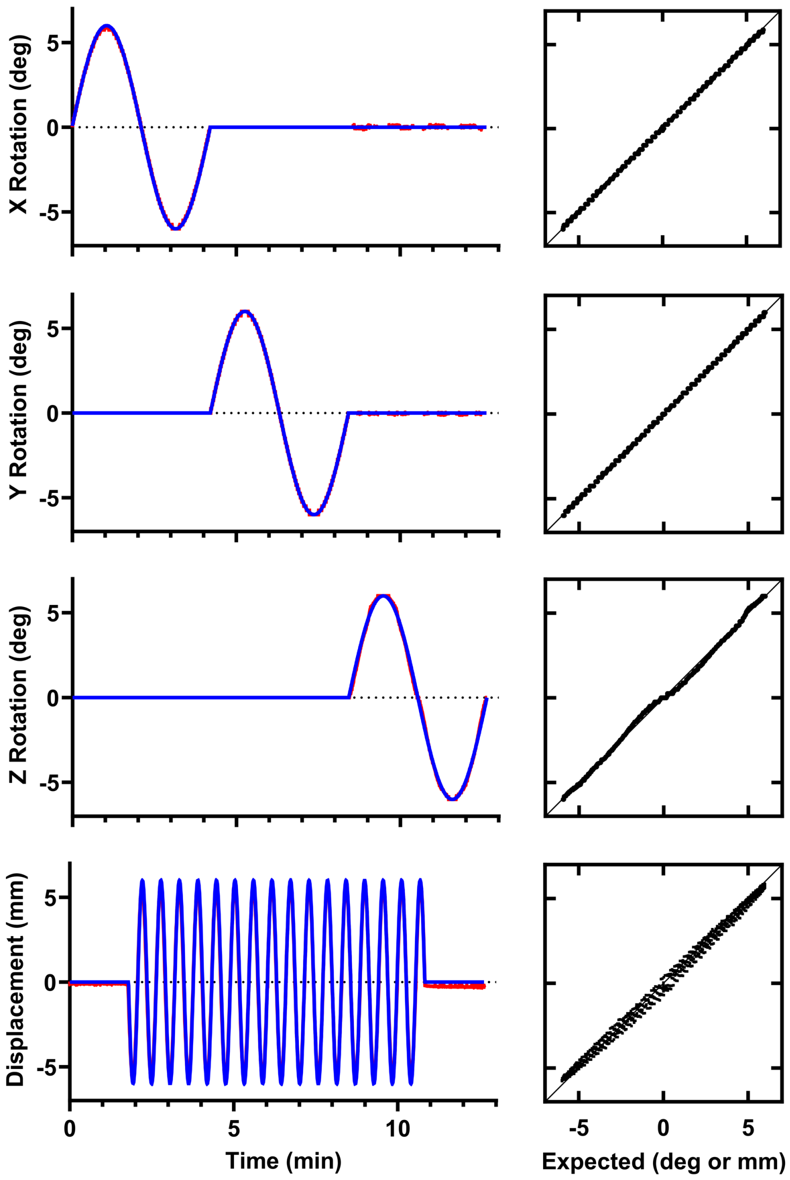

Supporting Information Figure S1. Plots of expected (blue) and measured (red) trajectories of rotation and displacement from phantom experiments with FID-based field correction of SNAV data. The same data is shown on the right in the form of calibration plots.

S2. Magnitude image quality quantified using NRMSE

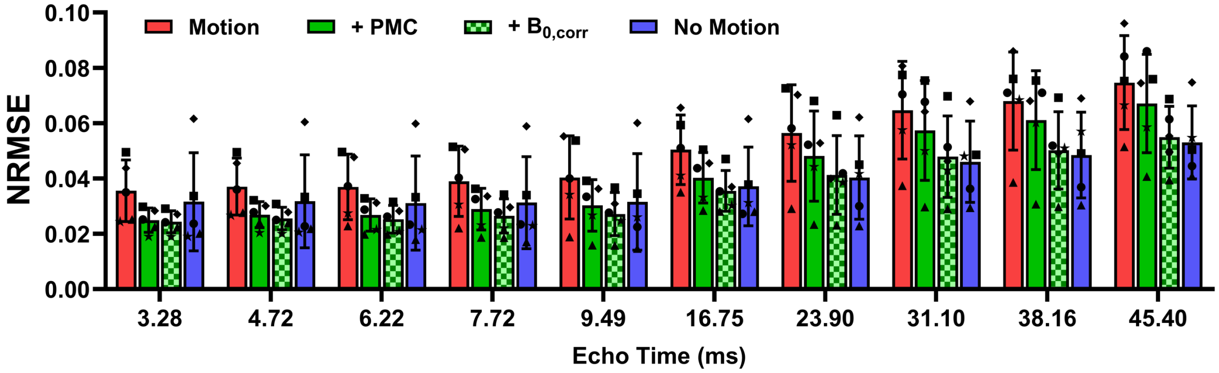

Supporting Information Figure S2. Magnitude image quality (quantified using NRMSE) as a function of echo time for all participants for cases of motion no correction, motion with PMC with and without additional retrospective field correction. Also shown are the results for a no motion repetition, meant to provide a reasonable measure of error which can be attributed to interscan variability. However, at least one such case (Case 3, denoted by the diamond symbols) demonstrated inflated error with no clear motion artifacts due to interscan variations in low-spatial-frequency signal inhomogeneity (attributed to motion-induced coil-sensitivity fluctuations relative to the reference acquisition). NRMSE, normalized RMSE.

S3. Minor motion case

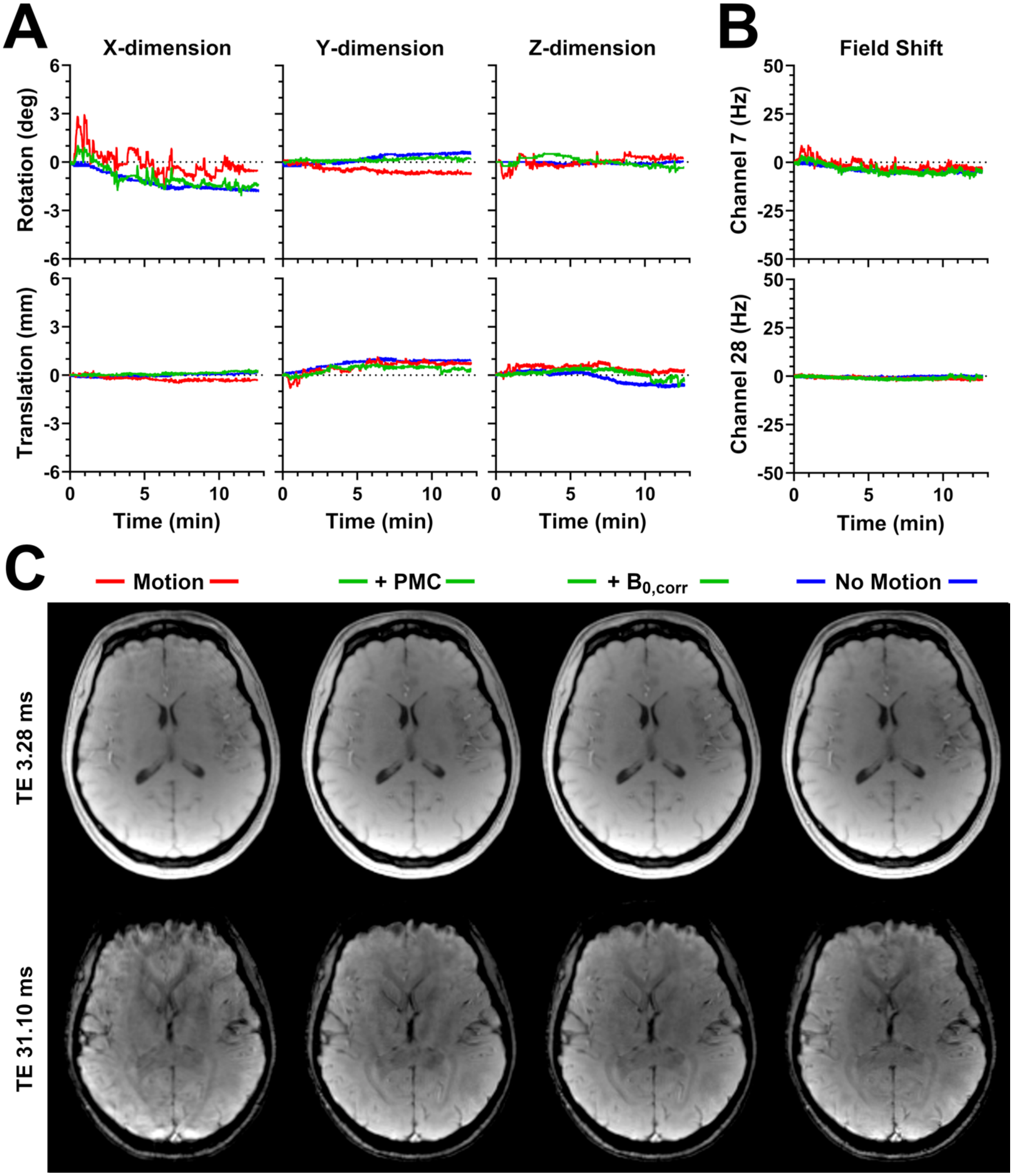

Supporting Information Figure S3. Measured (A) motion traces and (B) field shifts (shown for two sample channels) for a minor motion case (Case 1). (C) Sample magnitude images acquired with motion no correction, motion with PMC, additional retrospective field correction, and the no motion reference (shown for echoes one and eight).

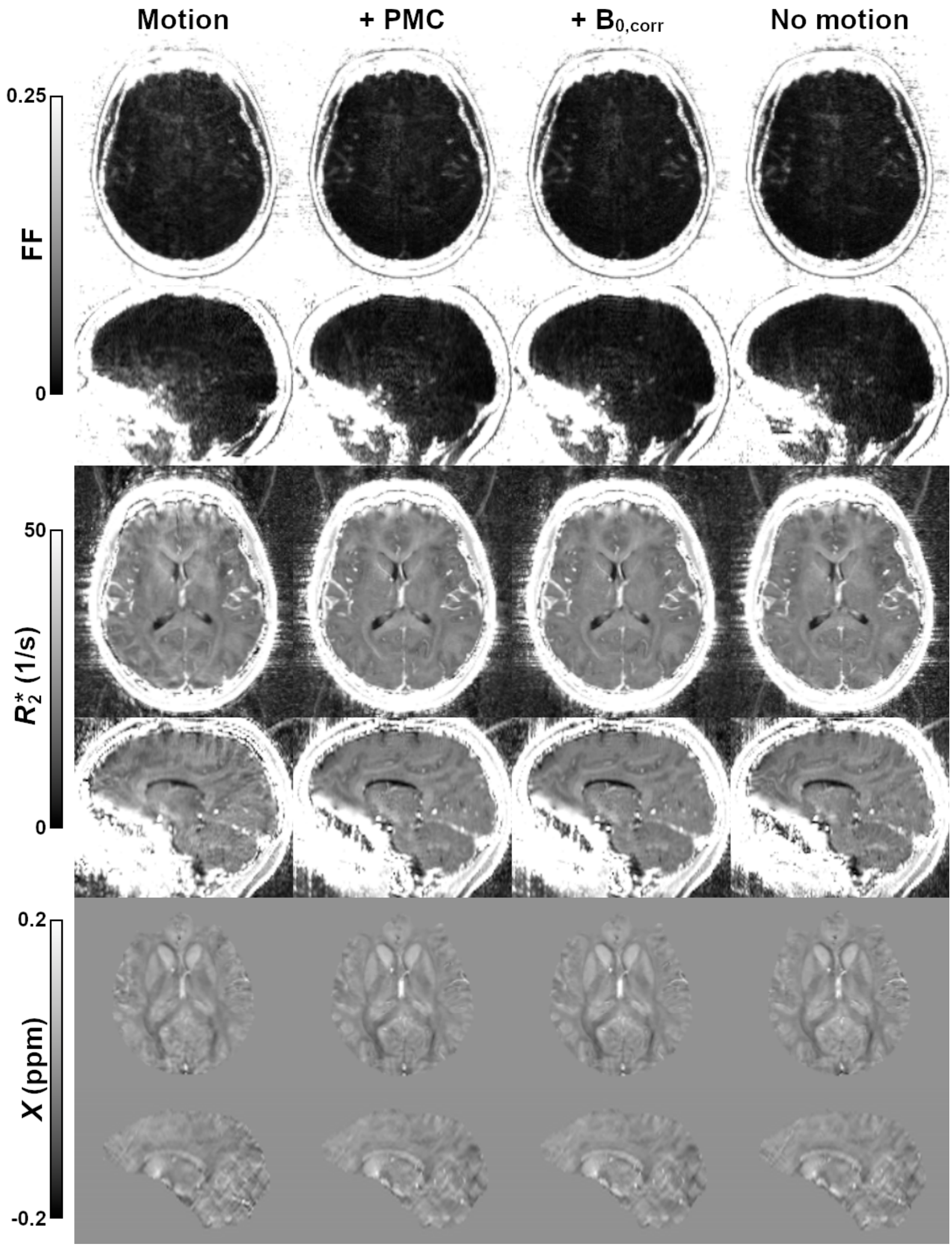

Supporting Information Figure S4. Axial and sagittal views of fat fraction, R2*, and susceptibility for Case 1 (minor motion).
